# A Pilot Study on the ^1^H-NMR Serum Metabolic Profile of Takotsubo Patients Reveals Systemic Response to Oxidative Stress

**DOI:** 10.3390/antiox10121982

**Published:** 2021-12-13

**Authors:** Domitilla Vanni, Nicola Viceconte, Greta Petrella, Flavio Giuseppe Biccirè, Francesco Pelliccia, Gaetano Tanzilli, Daniel Oscar Cicero

**Affiliations:** 1 Department of Chemical Science and Technology, University of Rome “Tor Vergata”, 00133 Rome, Italy; vanni@scienze.uniroma2.it (D.V.); petrella@scienze.uniroma2.it (G.P.); 2 Department of Internal Medicine, Anesthesiologic and Cardiovascular Sciences, Sapienza University of Rome, Policlinic Umberto I, 00161 Rome, Italy; nicola.viceconte@uniroma1.it (N.V.); flaviogiuseppe.biccire@uniroma1.it (F.G.B.); francesco.pelliccia@uniroma1.it (F.P.); gaetano.tanzilli@uniroma1.it (G.T.)

**Keywords:** Takotsubo syndrome, metabolomics, ^1^H-NMR, systemic stressor response

## Abstract

Takotsubo syndrome (TTS) presents as an acute coronary syndrome characterized by severe left ventricular (LV) dysfunction and non-obstructive coronary artery disease that typically shows spontaneous recovery within days or weeks. The mechanisms behind TTS are mainly related to beta-adrenergic overstimulation and acute endogenous catecholamine surge, both of which could increase oxidative status that may induce further deterioration of cardiac function. Although several studies reported evidence of inflammation and oxidative stress overload in myocardial tissue of TTS models, systemic biochemical evidence of augmented oxidant activity in patients with TTS is lacking. In this study, serum samples of ten TTS patients and ten controls have been analyzed using ^1^H-NMR spectroscopy. The results of this pilot study show a marked alteration in the systemic metabolic profile of TTS patients, mainly characterized by significant elevation of ketone bodies, 2-hydroxybutyrate, acetyl-L-carnitine, and glutamate levels, in contrast with a decrease of several amino acid levels. The overall metabolic fingerprint reflects a systemic response to oxidative stress caused by the stressor that triggered the syndrome’s onset.

## 1. Introduction

Few diseases show a more creative name and a more emotionally related origin than the Takotsubo syndrome (TTS), a disorder that differentially affects women. They account for 80% to 100% of cases, with a mean age at diagnosis of 61 to 76 years [1]. Aproximately 5–6% of women presenting suspected acute ST-segment elevation myocardial infarction (STEMI) suffer from TTS instead [2]. Japanese investigators named it in 1990 based on the similarity between the resulting left ventricle shape and an octopus pot with a round bottom and narrow neck traditionally used in their country [3].

TTS is often precipitated by major emotional/physical stress such as grief, fear/panic, anger, anxiety, embarrassment, or even positive emotions. For this reason, it has often been called “stress cardiomyopathy” or “broken heart syndrome”. Until recently, many cases of TTS were misdiagnosed as “myocardial infarction” (MI). A striking increase in apparently MI episodes was noticed in the past, coinciding with natural disasters such as earthquakes [4] or during major sporting events [5]. It was not until more recent times that it was possible to understand that the boosts were correlated to the underlying stress [6]. The diagnosis of TTS is complicated by the similarity of its clinical features to those of acute coronary syndrome, myocarditis, and spontaneous coronary artery dissection [7].

However, in the acute phase, patients with TTS have increased concentrations of plasma catecholamines (i.e., epinephrine, norepinephrine, and dopamine) and stress-related circulating neuropeptides that are several times higher than those in patients with myocardial infarction and remain markedly elevated even a week after the onset of symptoms [8]. Adrenergic overstimulation leads to LV dysfunction by direct myocardial cell injury and/or epicardial and microvascular coronary vasoconstriction [9]. On the other hand, it has been reported that the vast activation of adrenergic receptors may induce an increased production of reactive oxygen species (ROS) [10]. The high oxidative status may result in further myocardial cell damage and endothelial dysfunction that affects the production of vasoactive substances, thus impairing tissue perfusion at the microcirculatory level [11].

From a metabolic point of view, mental stress increases the concentration of oxidants in the CNS and peripheral organs [12], leading to dose-dependent changes in the hypothalamic–pituitary–adrenal (HPA) axis, the sympathetic nervous system (SNS), and the immune system [13]. Given the ostensible relationship of TTS with stressful situations, it is expected that alterations responding to oxidative stress will be a metabolic signature of patients with this disease.

Metabolomics represents an efficient tool to obtain insights into cardiovascular pathophysiology and to help in biomarker discovery, as already proved in the case of heart failure (HF), atherosclerosis, and ischemic and non-ischemic cardiomyopathy [14]. Novel strategies based on ^1^H NMR profiling of serum for the personalized diagnosis and prognosis of cardiovascular diseases have been developed. They can boost the development of personalized medicine, with the additional advantage of representing non-invasive methods capable of stratifying the disease risk at a population level [15]. On the contrary, little is known about the systemic metabolic alterations provoked by TTS. In a recent study, a limited set of four metabolite levels and the total lipid concentration were used to differentiate the metabolic profile of TTS patients from acute myocardial infarction, and between acute and subacute phases of the disease [16]. We used a much larger set of 53 metabolites and three ratios to differentiate the serum metabolic profiles of TTS and control groups using ^1^H-NMR spectroscopy. Each group was formed by ten subjects, which is generally a low number for a clinical study. However, it represents the average number of patients with TTS that a single institution receives in one year [17]. We hypothesized that the observed alterations were associated with oxidative stress’s systemic status. Furthermore, we discovered a correlation between serum level changes and cardiac function, suggesting that new metabolic markers can be added in the future to clinical practice improving diagnosis and prognosis of the disease.

## 2. Materials and Methods

### 2.1. Venous Serum Collection

Blood samples were collected following the ethical guidelines, and patients provided written consent. A 10 mL sample of venous blood was collected from each patient in nonfasting condition upon admission. All patients were medicated with aspirin and/or beta-blockers at baseline. After collection, the whole blood coagulated. The clothing was removed by centrifugation at 3000× *g* for 10 min in a refrigerated centrifuge. The resulting supernatant (serum) was stored at −80 °C.

### 2.2. Sample Preparation

Serum samples were prepared as previously described [18]. Briefly, samples were thawed and ultrafiltered to remove proteins using 3 kDa cutoff Amicon Ultra-0.5 centrifugal filter devices. The filters were washed four times with distilled water to remove glycerol (13,800× *g*, 4 °C for 20 min), then the samples were centrifuged at 13,800× *g*, 4 °C for 90 min. To each filtered serum, NMR buffer (250 mM phosphate buffer KH_2_PO_4_/K_2_HPO_4_, pH 7.4 containing 0.63 mM sodium trimethylsilyl propanoate-d_4_, 10% D_2_O, and 2% NaN_3_) was added to reach a final volume of 600 μL.

### 2.3. ^1^H-NMR Spectroscopy

All ^1^H-NMR spectra were acquired using a Bruker Avance 700 MHz spectrometer equipped with a triple resonance TXI probe and a SampleXpress Lite autosampler. All spectra were acquired at 25 °C using noesypr1d sequence, 1024 scans, 4 dummy scans, a spectral width of 16 ppm, an acquisition time of 3 s, a relaxation delay of 3 s, and a mixing time of 100 ms.

All the spectra were processed using 0.5 Hz of line-broadening followed by manual phase and baseline correction. Chenomx NMRSuite 8.5 (Chenomx Inc., Edmonton, AB, Canada) was used to quantify the metabolites. The spectra database in this software allows a manual deconvolution of the different signals and determines the concentration of the compounds that form the mixture. TSP was set as an internal standard, and 53 metabolites were quantified in almost all samples (missing values 5%). Concentration values of pyroglutamate were summed to those of glutamine as the cyclization reaction of glutamine to pyroglutamate occurs during protein removal [19].

### 2.4. Data Analysis

For univariate and multivariate analyses, metabolite concentrations were normalized according to the method of probabilistic quotients [20], while for the comparison with normal ranges this normalization was not perfomed. Multivariate data analysis was carried out using SIMCA-P (version 17.0.1. Umetrics AB, Umea, Sweden). All data were log-transformed and unit-variation scaled [21]. Classification models were constructed using orthogonal PLS modeling discriminant analysis (OPLS-DA) [22]. Metabolite concentrations, measured using Chenomx, three ratios, and the total amino acids concentration were used as variables. The following parameters evaluated the robustness of the models: R^2^Y, predicted percentage of the response; R^2^X, variation of X explained by the model; and Q^2^, the goodness of prediction. R^2^ varies between 0 and 1, Q^2^ varies between −1 and 1. When the Q^2^ value is higher than 0.5, the predicted model is good [23]. In the models, the influence on Y variation of every variable (called variable importance in the projection (VIP)) was used to consider which metabolites are involved in the supervised analysis. Moreover, ANOVA of the cross-validated residual (CV ANOVA) and permutation tests were performed to assess the significance of multivariate models. All these parameters were calculated using SIMCA.

### 2.5. Minimum Sample Size Calculation

The minimum sample size was calculated using the following equation [24]:

(1) $$n=\frac{{2\sigma }^{2}{\left(Z_{\beta }+Z_{\alpha }\right)}^{2}}{{∆}^{2}}$$

where *n* is the minimum sample size per group; σ is the population variance; Z_β_ is the conventional multiplier for power = 0.80 (0.84); Z_α_ is the conventional multiplier for α = 0.05 (1.96); ∆^2^ is the difference between the population means.

## 3. Results

### 3.1. Serum Metabolic Profile Differentiate Takotsubo from Control Subjects

This pilot study enrolled one male and nine female patients showing TTS and ten control subjects (CTRL). Their baseline clinical characteristics are shown in Table 1. Venous serum samples of TTS patients were collected just after their recovery. The two main comorbidities were diabetes (20%) and hypertension (40–60%), but no significant difference exists between the two groups. On the contrary, the left ventricular ejection fraction (LVEF) percent is significantly lower for the TTS group.

**Figure 1:**
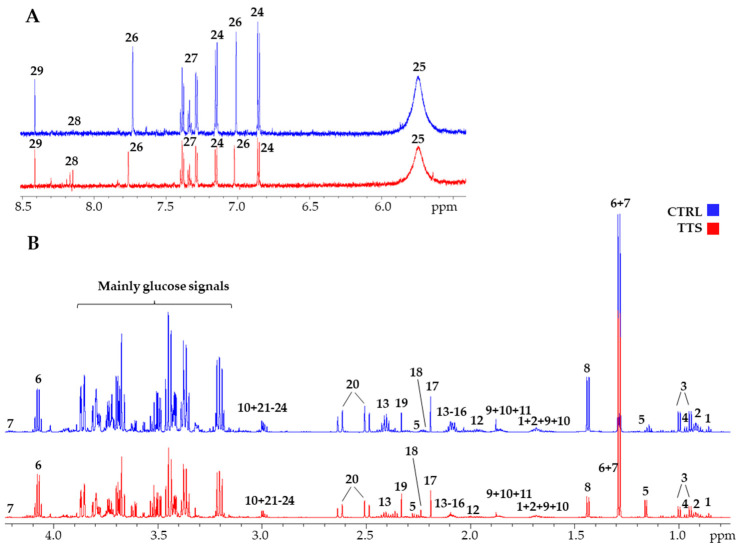
700 MHz ^1^H-NMR spectra of a TTS patient (red line) and a control (blue line). (**A**): aromatic portion; (**B**): aliphatic portion. The scale is set to the same internal standard concentration. 1: 2-hydroxybutyrate; 2: Leucine; 3: Valine; 4: Isoleucine; 5: 3-hydroxybutyrate; 6: Lactate; 7: Threonine; 8: Alanine; 9: Arginine; 10: Lysine; 11: Acetate; 12: Proline; 13: Glutamine; 14: Glutamate; 15: Methionine; 16: Acetyl-L-carnitine; 17: Acetone; 18: Acetoacetate; 19: Pyruvate; 20: Citrate; 21: Creatinine; 22: Creatine; 23: Ornithine; 24: Tyrosine; 25: Urea; 26: Histidine; 27: Phenylalanine; 28: Hypoxanthine; 29: Formate.

Figure 1 shows a typical 700 MHz ^1^H-NMR spectra of serum metabolites with the principal signals of the quantified compounds highlighted, both for a control subject and a TTS patient. A total of 53 metabolites were identified and quantified by spectral deconvolution using the software Chenomx as already described [18]. Their median concentration values and the chemical shift of the signals used for their quantification are shown in Supplementary Material Table S1. In addition, the total amino acid concentration and three ratios were calculated, namely acetoacetate/3-hydroxybutyrate (AcAc/3HB), acetyl-L-carnitine/L-carnitine (ALCAR/CAR), and phenylalanine/tyrosine (Phe/Tyr) (Supplementary Material Table S2). These compounds cover most of the principal families of serum metabolites, including amino acids and their metabolites, carbohydrate metabolism, gut microbiota metabolites, fatty acids/lipids metabolism, ketone bodies (KB), nucleoside metabolism, energy metabolism, and TCA- and urea-cycle intermediates. They provide good coverage of the principal pathways that can be altered in TTS, and their concentrations were used to construct a metabolic profile to be compared with that of the control group.

**Figure 2:**
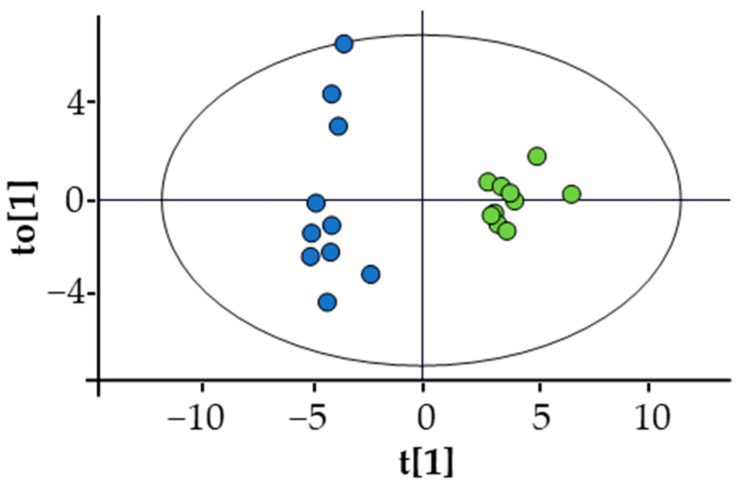
Score plot of the OPLS-DA of TTS (blue dots) vs. CTRL (green dots). Statistical parameters: R^2^X(cum) = 0.413; R^2^Y(cum) = 0.949; Q^2^(cum) = 0.851; CV ANOVA = 4.7 × 10^−6^. Permutation test was also performed: number of permutations = 100; R^2^ intercept = 0.643; Q^2^ intercept = −0.334. None of the permutated models showed higher R^2^ or Q^2^.

**Figure 3:**
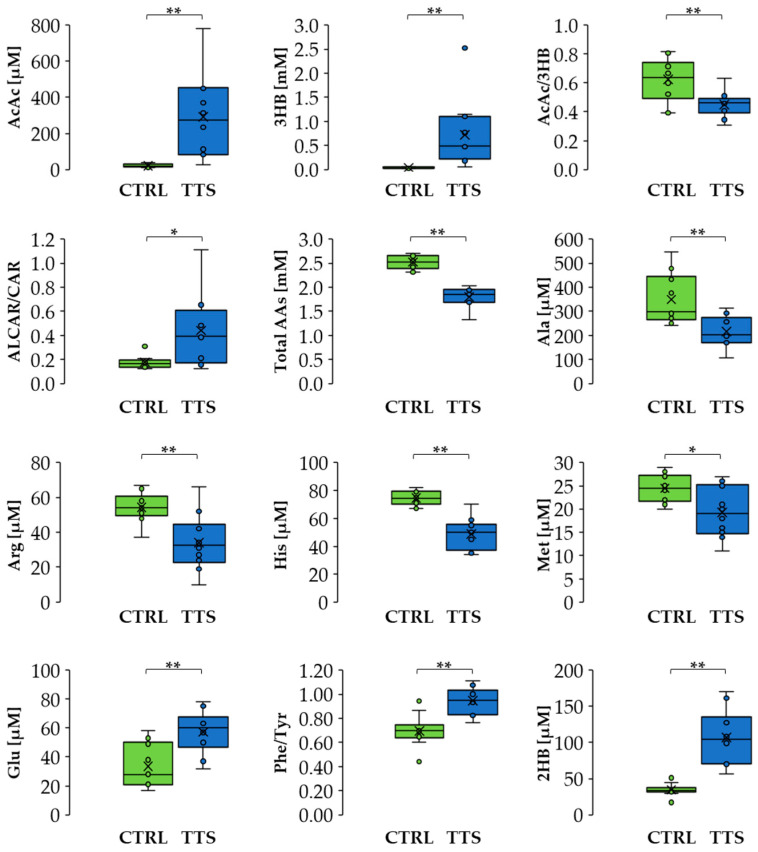
Differences in metabolite levels and ratios between CTRL and TTS. Boxes denote IQR, lines denote median, whiskers denote the 5th and 95th percentile, and x denotes the average. * *p* < 0.05, ** *p* < 0.01. Abbreviation used: AcAc: acetoacetate; 3HB: 3-hydroxybutyrate; ALCAR: acetyl-L-carnitine; CAR: L-carnitine; Ala: alanine; Arg: arginine; His: histidine; Met: methionine; Glu: glutamate; Phe: phenylalanine; Tyr: tyrosine; 2HB: 2-hydroxybutyrate.

A multivariate analysis using an OPLS-DA model (Figure 2) showed significant separation between the profiles of the two groups. The list of metabolites, including the ratios AcAc/3HB, ALCAR/CAR, and Phe/Tyr, that mainly contributed to this difference can be found in Supplementary Material Table S3. It includes amino acids, which were generally decreased in TTS patients except for glutamate that showed an increase, metabolites of the branched-chain amino acids (BCAA), glycerol, trimethylamine N-oxide (TMAO), KB, 2-aminobutyrate, 2-hydroxybutyrate, and uridine. In addition, the TTS profile showed an increase in the ALCAR/CAR ratio and a decrease in the Phe/Tyr and AcAc/3HB ratios. Notably, the total amino acid concentration was lower in TTS patients with respect to the control group. Figure 3 shows the variation of selected metabolites and ratios contributing to the discrimination between the two metabolic profiles.

We have then calculated the minimum sample size that can statistically sustain our results, given the magnitude of the observed differences [24]. As a result, we found that for ten out of the twenty-eight significantly altered metabolites and ratios, the minimum sample size for each group was between six and ten, in line with our cohort sizes (Supplementary Material Table S3).

### 3.2. Ketone Bodies, Fatty Acid Metabolism, and Amino Acid Levels Are Unbalanced in Takotsubo Patients with Respect to Normal Values

**Figure 4:**
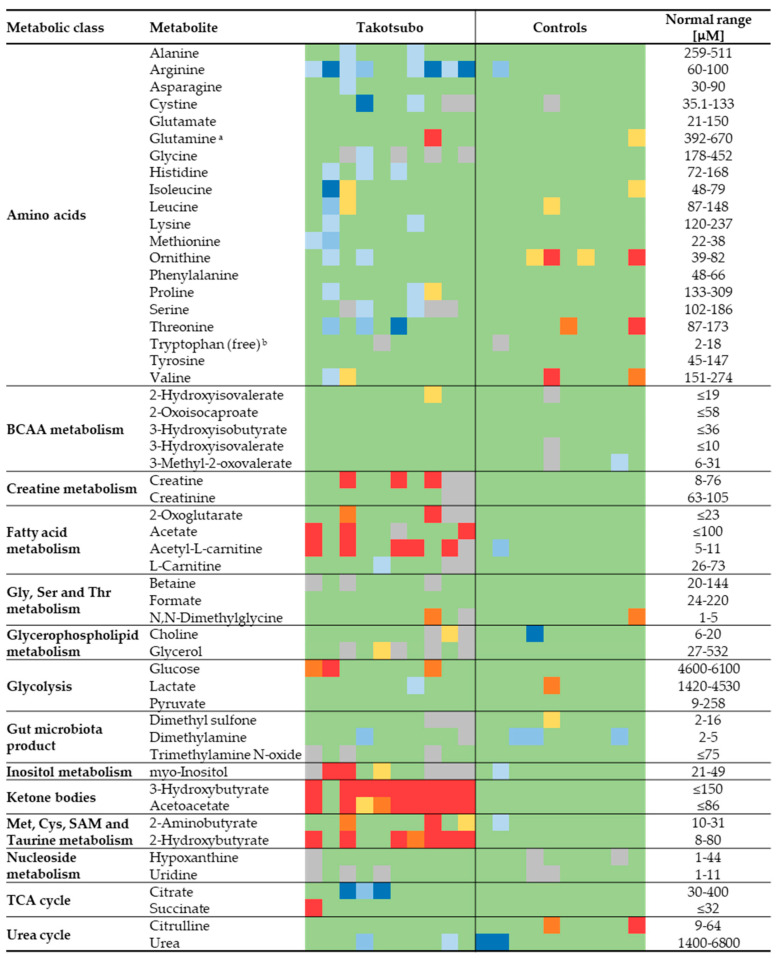
Heatmap showing the general agreement between the measured value for 53 metabolites in each TTS and CTRL sample and the ranges found in the literature. Values in green lie within the range or exceed less than 20% of the limits; in yellow, orange, and red, those that exceed 20, 35 and 50% of the maximum, respectively; in blue shades, those lower than 20, 35 and 50% of the minimum. Cells in grey represent missing values. ^a^ Sum of glutamine and pyroglutamate (see Section 2). ^b^ The normal range refers to the fraction unbound of tryptophan in human blood [27].

The absolute concentrations measured by NMR provide the opportunity to use as control the reference ranges reported in the literature, thereby allowing for an extra validation of the results. The reference values were obtained mainly through the Serum Metabolome server [25], containing information about 4651 small molecule metabolites found in human serum and 10,895 concentration values [26]. Figure 4 shows a heatmap to compare all the metabolite concentrations quantified in this study for both groups with the literature-derived values. The concentrations obtained for the control group resulted within the normal range in the vast majority of cases. A very different picture emerged for TTS patients, which generally show lower than normal amino acid levels, higher levels of metabolites of the fatty acid metabolism (mainly acetate and ALCAR), KB, and 2-hydroxybutyrate.

### 3.3. The Metabolic Profile Correlates Significantly with the Left Ventricular Ejection Fraction

**Figure 5:**
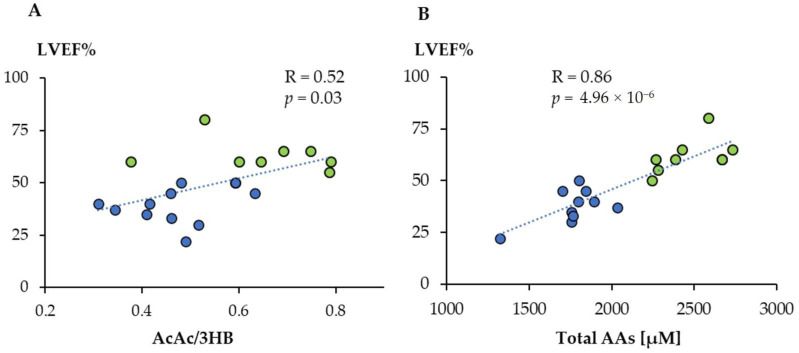
Linear correlation between acetoacetate/3-hydroxybutyrate ratio (AcAc/3HB) (**A**) and total amino acids concentration (**B**) with LVEF%. Green dots: controls; blue dots: TTS.

We then wanted to determine whether there was a correlation between the metabolic profiles obtained by ^1^H-NMR and LVEF%, one of the most used parameters related to the prognosis of cardiovascular diseases, and that was significantly lower for the TTS group. We found a significant linear correlation between LVEF% values and the AcAc/3HB ratio (Figure 5A). Moreover, the total amino acid concentration showed an even higher association, indicating that its value can be used to anticipate the level of LVEF (Figure 5B). We have then searched those metabolites that displayed a significant linear correlation with LVEF% (Supplementary Material Table S4). They included eight amino acids positively and six other metabolites negatively correlated. Among these last compounds, there are different metabolic products of amino acids such as 2-oxoglutarate (2OG), 2-Oxoisocaproate (2OIC), 3-Methyl-2-oxovalerate (3M2OV), and 2HB. In addition, we found significant negative correlations for myo-inositol and urea.

Taking all together, these results indicate that the serum metabolic profile allows the distinction between TTS and control subjects. The multivariate analysis and the comparison with normal values from literature show a decrease in the amino acid levels and a significant increase in circulating KB, 2-hydroxybutyrate, and fatty acid metabolism-related markers. In addition, there is a correlation between the levels of various metabolites and cardiac function, as measured by the LVEF% value.

## 4. Discussion

A significant perturbation of the serum metabolic profile was observed for TTS patients, both in comparison with an age and sex-matched control group and with respect to normal values of the literature, measured under a wide range of factors including age, gender, genetic background, diurnal variation, health status, activity level, and diet. The last analysis is particularly relevant since it uses a large number of observations as a reference and can be used to build a personalized metabolic profile, similar to that already proposed for urine [28]. Moreover, the results depicted in Figure 4 are more easily translatable into normal clinical practice, possibly enhancing the number of features that can be used to characterize the disease.

**Scheme 1:**
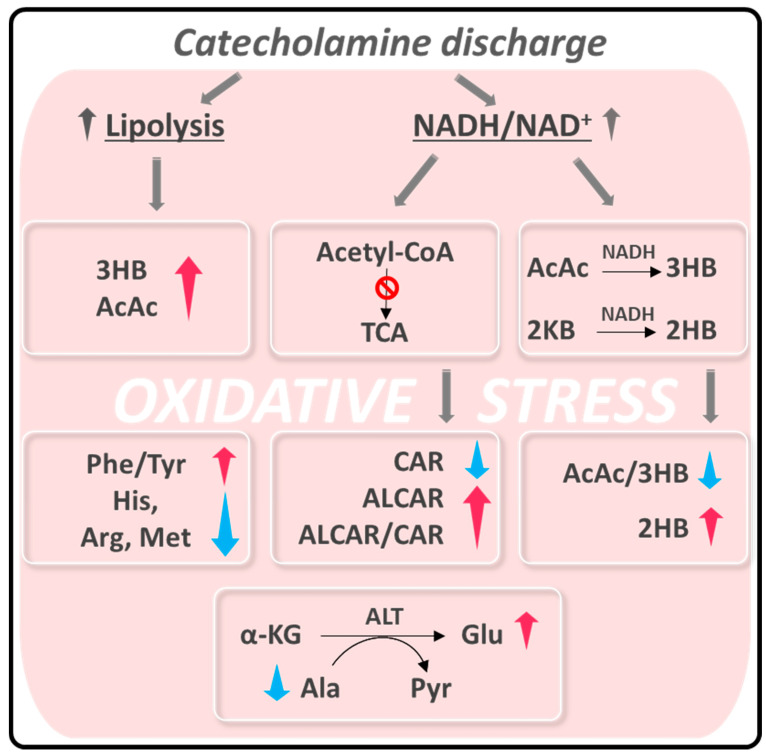
Summary of alteration observed in TTS patients related to oxidative stress. Abbreviations are the same as in Figure 3. 2KB: α-ketobutyrate; α-KG: α-ketoglutarate; ALT: alanine aminotransferase; Pyr: Pyruvate.

One of the most accepted hypotheses about the origin of acute episodes of TTS puts in play a pulse exposure of catecholamines based on the observation that plasma levels of epinephrine, norepinephrine, and dopamine were elevated in the acute phase of TTS in nearly 75% of patients [8]. This elevation prompts both vascular inflammatory conditions at a myocardial level [29,30], increasing ROS production and finally causing coronary endothelial dysfunction [2]. On the other hand, it is known that exposure to intrinsic or extrinsic stressors compromises the cellular redox homeostasis of the entire organism, resulting in a preponderance of oxidants [31]. Systemic oxidative stress may also contribute to the inflammatory response associated with TTS [32]. This hypothesis fits with the changes in the serum metabolic profile of TTS patients observed in our study, which we have interpreted as a response to this redox unbalance (Scheme 1).

The catecholamine discharge and the consequent oxidative stress provoke two main metabolic alterations: (i) enhanced lipolysis [33,34,35] and (ii) a rise in the intracellular NADH/NAD^+^ ratio [36,37].

Catecholamines stimulate lipolysis in adipose tissue, incrementing the level of circulating free fatty acids (FFAs) [35]. As blood KB levels correlate with circulating norepinephrine and FFAs, the enhanced lipolysis provokes ketonemia, coherent with the significant increment we observed for TTS patients. We found values higher than normal for nine TTS patients, reaching a maximum of almost 3 mM in 3HB and 1 mM in AcAc. In contrast, healthy subjects show concentrations of KB as low as 19 μM, which can increase to approximately 120 μM after an overnight fast [38]. Our TTS patients were studied in the nonfasting state upon admission, thereby enhancing the significance of the high KB levels observed.

The KB levels measured in our study are similar to those of a recent study about the circulating KB in humans after ST-segment elevation myocardial infarction (STEMI), which demonstrated for the first time that KB concentrations were >3-fold higher in patients with this condition, reporting values up to 8 mM in 3HB and 1 mM in AcAc [39]. This large increase was related to the systemic catecholamine surge and free fatty acid release produced to respond to myocardial infarction (MI) [39]. On the other hand, a decrease in 3HB and AcAc was measured in patients with HF showing a reduced ejection fraction and unchanged in those with preserved ejection fraction [40].

KB represent fundamental substrates for the heart and brain when glucose availability is reduced, and their utilization is directly related to their systemic concentration. In addition to its use as energy fuel, 3HB also exerts signaling roles in maintaining energy homeostasis and promoting oxidative stress resistance [35,41]. 3HB acts as a direct scavenger of hydroxyl radical OH^•^, inhibits inflammatory intermediates synthesis and mitochondrial ROS production, and promotes antioxidant pathways through the regulation of FOXO1, FOXO3, and NRF2 transcription factors [34,40,41].

An increase in ROS levels causes an intracellular elevation of the NADH/NAD^+^ ratio, particularly in the liver and cardiac muscle [36,37]. Excess NADH is redirected to the conversion of AcAc to 3HB [42,43], explaining the drop in the AcAc/3HB ratio observed in the serum of TTS patients. The rise in 2HB concentration could also be related to the increase in NADH that would favor the reduction of 2-ketobutyrate (2KB) to 2HB [44].

Another consequence of the NADH concentration elevation is the inhibition of acetyl-CoA’s entry in the TCA cycle to stop its production [42,43]. Raised circulating ALCAR levels, as observed in TTS serum samples, could reflect a mismatch in acetyl-CoA formation from FA and glucose oxidation and its utilization in the TCA cycle [45]. In turn, the increase in ALCAR induces a decrease in the concentration of circulating CAR, causing a significant increment of the ALCAR/CAR ratio. Besides its function as an energy substrate, ALCAR also protects the tissues against oxidative stress [46,47,48].

We noticed an important unbalance in circulating amino acid levels in the metabolic profile of TTS patients. The total amino acid concentrations were significantly lower in the serum of TTS patients compared to the control group. Histidine, arginine, methionine, and alanine were among the amino acids that showed the most significant reductions. On the contrary, glutamate and the Phe/Tyr ratio were increased in TTS patients. All these changes can be related to a systemic response to oxidative stress. A decrease in serum histidine and arginine has been associated with inflammation and oxidative stress in obese women, while histidine was negatively associated with oxidative stress in non-obese controls [49]. Methionine is directly linked to oxidative stress, mainly through its catabolism products glutathione and taurine [50]. Hepatocytes readily catalyze the direct synthesis of glutathione from methionine as a response to an increase in ROS [51].

In addition, we can speculate the existence of a link between the opposite changes in circulating alanine and glutamate levels. An elevated serum level of alanine aminotransferase (ALT) was considered a potential marker of inflammation and oxidative stress [52]. Elevation of this enzyme activity, which catalyzes the transamination between alanine and α-ketobutyrate to form pyruvate and glutamate, causes a decrease in alanine and an increase in glutamate. ALT levels are routinely measured to diagnose liver disease, but more recently they were associated with cardiovascular disease (CVD) risk [53]. Correlation between high ALT and CVD or mortality is explained by underlying hepatic inflammation [53], a plausible scenario provoked by oxidative stress in TTS. Finally, an increase in the serum Phe/Tyr ratio was related to the inflammatory response [54] or a diminished phenylalanine hydroxylase activity, caused by its oxidation in an environment of high ROS concentration [55].

Our study revealed a significant correlation between the serum level of different metabolites and LVEF, the central measure of left ventricular systolic function. Its value shows a significant reduction during the acute phase of TTS [56,57], in line with our data (Table 1). A recent study estimated that an LVEF cutoff of 38% is associated with significantly worse survival [7]. However, LVEF alone is insufficient to assess cardiac functional improvement, and more sensitive markers are needed to further risk stratify. We have found a linear correlation of the total amino acid concentration and several metabolite levels with LVEF (Figure 5 and Supplementary Material Table S4). The direction of the former alteration seems to be a specific metabolic signature of TTS compared to HF, for which the total amino acid concentration was observed to increase [58] instead of the decrease observed for TTS.

Moreover, the search of the metabolic fingerprint of HF with preserved ejection fraction revealed that six amino acids were elevated with respect to a control group [40]. Finally, tryptophan-arginine-leucine were also increased among acute MI patients suggesting that the biosynthesis of amino acids can be used as an indicator for acute MI [59]. These results constitute additional hints that the decrease observed in our work constitutes a characteristic feature of the TTS systemic response.

Our study had several limitations. The first is the reduced number of subjects enrolled. Increasing this number will imply extending the time of patient recruiting and/or involving several institutes as in other studies [17]. Although our cohorts were limited in size (ten subjects per group), they were adequate to characterize the sizeable metabolic change accompanying TTS, as confirmed by the calculated minimum sample size (Table S3). The extension to a larger population is undoubtedly a requirement to ensure the results of this pilot experiment and would allow us to adjust for covariates such as past medical history or medications that may affect our results. The second refers to the fact that the outcomes of a metabolomics study cannot provide conclusive evidence on the origin of the observed metabolic alterations. In this type of study, we limit ourselves to the generation of hypotheses requiring future experiments to be reliably proven, including direct oxidative stress load assessment by determining the levels of catecholamines and reactive oxygen species such as H_2_O_2_. A final limitation of this research is that no subjects with other cardiomyopathies other than TTS, particularly STEMI, were included. However, some results could be compared with other studies indicating certain particular features in the metabolic profile of TTS patients, such as the decrease in the amino acid levels. Further studies are thus needed to investigate the specific alterations in global metabolism associated with TTS that can improve our understanding of the disease’s systemic consequences, opening new avenues for specific treatments.

## 5. Conclusions

The correlation between TTS and oxidative stress at the myocardial level is already well established [2]. Our results about the alteration in circulating metabolites highlight a possible generalization of oxidative stress to other tissues in line with the known systemic response to emotional stressors [31]. Consequently, the large increment in circulating KB, ALCAR, glutamate, and Phe/Tyr ratio, and the decrease in total amino acids, histidine, arginine, alanine, and methionine, can be explained as a response to the ROS increase at a systemic level. In addition, we found that the change in the metabolic profile is proportional to the severity of cardiac dysfunction because it correlates significantly with the LVEF value. Based on this result, we can envisage that the future inclusion of metabolic markers in clinical practice might improve risk stratification in TTS.

## Figures and Tables

**Table 1:** Baseline characteristics of control and Takotsubo groups.

	CTRL *n* = 10	TTS *n* = 10
Age ^a^	57 (38–72)	68 (40–84)
Female sex %	100	90
Diabetes %	20	20
Hypertension %	40	60
Smoking %	30	10
LVEF % ^a,b^	62 (50–80)	38 (22–50)

^a^ average and range indicated. ^b^ left ventricular ejection fraction.

## Data Availability

The data presented in this study are available in article and supplementary material.

## Supplementary Materials

The following are available online at https://www.mdpi.com/article/10.3390/antiox10121982/s1, Table S1: List of the 53 metabolites and total amino acid concentration measured in control and TTS groups, Table S2: Median and interquartile range (IQR) for control and TTS groups of the calculated ratios, Table S3: Fold changes, statistical parameters, and minimum sample size of the metabolites contributing to the separation between the TTS and control metabolic profiles, Table S4: Metabolites showing the highest correlation with LVEF%.
