# Supplementary material for: A Pilot Study on the 1H-NMR Serum Metabolic Profile of Takotsubo Patients Reveals Systemic Response to Oxidative Stress
Source: Antioxidants (Basel). 2021 Dec 13;10(12):1982. doi: 10.3390/antiox10121982 (PMC8750825; doi:10.3390/antiox10121982)
Supplement: Supplementary file 1 [file antioxidants-10-01982-s001.zip › antioxidants-1508944-supplementary.pdf]

Table S1. List of the 53 metabolites and total amino acid concentration measured in control and TTS groups. The chemical shifts used for the quantification and the median, interquartile range (IQR), and the number of spectra in which each metabolite was quantified (N) are shown.

Metabolite	δ [ppm][a]	Controls Median [μM] (IQR), N	Takotsubo Median [μM] (IQR), N
**2-Aminobutyrate**	1.0	24 (14-26), 10	30 (25-34), 10
**2-Hydroxybutyrate**	0.9	45 (41-45), 10	141 (106-166), 10
**2-Hydroxyisovalerate**	0.8	6 (5-7), 9	10 (7-12), 10
**2-Oxoglutarate**	3.0	14 (12-16), 10	21 (15-26), 8
**2-Oxoisocaproate**	2.6	5 (4-7), 10	14 (11-16), 10
**3-Hydroxybutyrate**	1.2	33 (29-58), 10	655 (326-1324), 10
**3-Hydroxyisobutyrate**	1.1	16 (16-18), 10	24 (19-31), 10
**3-Hydroxyisovalerate**	1.3	1 (1-2), 9	3 (2-3), 10
**3-Methyl-2-oxovalerate**	1.1	6 (6-7), 9	12 (10-13), 10
**Acetate**	1.9	60 (51-87), 10	73 (47-152), 9
**Acetoacetate**	2.3	22 (18-37), 10	369 (126-559), 10
**Acetyl-L-carnitine**	3.2	8 (6-8), 10	17 (7-18), 9
**Alanine**	1.5	391 (354-534), 10	259 (234-366), 10
**Arginine**	1.6	69 (64-79), 10	42 (35-54), 10
**Asparagine**	2.8; 2.9	59 (56-66), 10	44 (36-61), 10
**Betaine**	3.9	30 (27-44), 10	26 (26-34), 7
**Choline**	3.2	11 (9-12), 10	10 (8-12), 8
**Citrate**	2.7	116 (95-138), 10	80 (29-125), 10
**Citrulline**	1.5	42 (37-57), 10	34 (23-44), 10
**Creatine**	3.0; 3.9	49 (42-56), 10	87 (57-96), 8
**Creatinine**	3.0; 4.0	77 (74-84), 10	83 (79-86), 8
**Cystine**	4.1; 3.2	53 (43-55), 9	52 (44-59), 8
**Dimethyl sulfone**	3.1	10 (7-11), 10	8 (6-14), 7
**Dimethylamine**	2.7	2 (1-2), 10	2 (2-3), 9
**Formate**	8.4	51 (42-55), 10	40 (32-55), 10
**Glucose**	3.9; 3.5	5370 (5125-5755), 10	6280 (5509-7162), 10
**Glutamate**	2.3	37 (29-60), 10	82 (62-92), 10
**Glutamine**[b]	2.5	715 (699-791), 10	560 (519-620), 10
**Glycerol**	3.6; 3.6	257 (223-297), 10	544 (349-603), 6
**Glycine**	3.5	294 (277-314), 10	249 (220-279), 6
**Histidine**	7.1	95 (90-104), 10	70 (54-74), 10
**Hypoxanthine**	8.2; 8.2	7 (5-8), 8	5 (4-6), 9
**Isoleucine**	1.0	61 (57-68), 10	63 (61-70), 10
**L-Carnitine**	3.2	47 (40-51), 10	42 (32-46), 8
**Lactate**	1.3	2533 (2393-3437), 10	1988 (1458-2443), 10
**Leucine**	1.0; 0.9	127 (126-133), 10	131 (111-144), 10
**Lysine**	3.0	212 (188-224), 10	126 (120-147), 10
**Methionine**	2.6	32 (29-34), 10	26 (21-30), 10
**myo-Inositol**	4.1; 3.6; 3.3	29 (24-33), 10	42 (31-60), 6
**N,N-Dimethylglycine**	2.0	3 (2-5), 10	4 (3-5), 9
**Ornithine**	3.0	85 (71-107), 10	49 (41-50), 10
**Phenylalanine**	7.4; 7.4; 7.3	56 (55-63), 10	58 (51-65), 10
**Proline**	3.3	230 (218-235), 10	178 (151-247), 10
**Pyruvate**	2.4	84 (67-122), 10	113 (77-164), 10
**Serine**	3.9	134 (128-142), 10	108 (92-131), 7
**Succinate**	2.4	7 (7-8), 10	12 (9-17), 10

**Threonine**	4.2; 3.6	142 (139-183), 10	89 (70-116), 10
**Trimethylamine N-oxide**	3.3	15 (14-19), 10	7 (6-14), 7
**Tryptophan (free)[c]**	7.7; 7.5	6 (5-8), 9	8 (7-10), 9
**Tyrosine**	7.2	85 (83-91), 10	61 (58-68), 10
**Urea**	5.8	1667 (1370-1973), 10	2086 (1654-2888), 10
**Uridine**	5.9	5 (5-6), 8	4 (3-5), 7
**Valine**	1.0	267 (250-302), 10	238 (213-271), 10
**Total amino acids**	-	3147 (3061-3332), 10	2450 (2273-2497), 10

[a] chemical shifts refer to TSP signal at 25°C.

[b]Sum of glutamine and pyroglutamate (see Materials and Methods)

[c] values of the fraction unbound of tryptophan

Table S2. Median and interquartile range (IQR) for control and TTS groups of the calculated ratios. AcAc/3HB: acetoacetate/3-hydroxybutyrate; ALCAR/CAR: acetyl-L-carnitine/L-carnitine; Phe/Tyr: phenylalanine/tyrosine.

Metabolite	Controls Median (IQR)	Takotsubo Median (IQR)
**AcAc/3HB**	0.62 (0.54-0.75)	0.46 (0.41-0.49)
**ALCAR/CAR**	0.17 (0.13-0.18)	0.37 (0.2-0.53)
**Phe/Tyr**	0.69 (0.65-0.71)	0.96 (0.86-1.02)

Table S3. Fold changes, statistical parameters, and minimum sample size of the metabolites contributing to the separation between the TTS and control metabolic profiles. AcAc/3HB: acetoacetate/3-hydroxybutyrate; ALCAR/CAR: acetyl-L-carnitina/L-carnitine; Phe/Tyr: phenylalanine/tyrosine; Total AAs: total amino acids concentration.

Metabolic class	Metabolite	FC	p-value	AUC	VIP	Minimum Sample size
Amino acids	Alanine	-0.31	<0.01	0.84	1.07	13
	Arginine	-0.31	<0.01	0.80	1.10	12
	Asparagine	-0.14	0.04	0.68	0.96	21
	Glutamate	0.87	<0.01	0.88	1.21	12
	Glutamine*	-0.13	<0.01	0.75	1.02	15
	Histidine	-0.28	<0.01	0.90	1.49	7
	Lysine	-0.31	<0.01	0.89	1.63	6
	Methionine	-0.13	0.02	0.65	1.03	18
	Ornithine	-0.43	<0.01	0.79	1.52	9
	Threonine	-0.40	<0.01	0.79	1.34	9
	Tyrosine	-0.23	<0.01	0.76	1.51	9
	Valine	-0.11	0.04	0.55	0.93	23
	Total amino acids	-0.20	<0.01	0.78	1.61	6
	Phe/Tyr	0.36	<0.01	0.94	1.49	10
BCAA metabolism	2-Hydroxyisovalerate	0.80	0.03	0.82	0.90	20
	2-Oxoisocaproate	1.96	<0.01	1.00	1.48	9
	3-Hydroxyisobutyrate	0.55	0.02	0.85	1.03	18
	3-Hydroxyisovalerate	0.67	<0.01	0.85	1.11	15
	3-Methyl-2-oxovalerate	1.11	<0.01	0.98	1.71	6
Fatty acid metabolism	ALCAR/CAR	1.53	0.02	0.65	0.98	17
Glycerophospholipid metabolism	Glycerol	0.74	0.03	0.55	1.31	13
Gut microbiota product	Trimethylamine N-oxide	-0.36	0.03	0.82	1.28	14
Ketone bodies	3-Hydroxybutyrate	20.4	0.02	0.96	1.49	15
	Acetoacetate	14.0	<0.01	0.96	1.44	12
	AcAc/3HB	-0.27	<0.01	0.81	1.15	14
Met, Cys, SAM and Taurine metabolism	2-Aminobutyrate	0.66	0.03	0.80	0.82	22
	2-Hydroxybutyrate	2.61	<0.01	1.00	1.64	8
Nucleoside metabolism	Uridine	-0.22	0.12	0.67	1.01	29

*Sum of glutamine and pyroglutamate (see Materials and Methods)

Table S4. Metabolites showing the highest correlation with LVEF%.

**Metabolite**	**Pearson *r***	**p-value**
Lysine	0.87	< 0.01
Histidine	0.83	< 0.01
Tyrosine	0.78	< 0.01
Methionine	0.73	< 0.01
Alanine	0.64	< 0.01
Threonine	0.63	< 0.01
Glutamine	0.58	< 0.05
Asparagine	0.52	< 0.05
2-Oxoglutarate	-0.53	< 0.05
2-Oxoisocaproate	-0.61	< 0.01
2-Hydroxybutyrate	-0.61	< 0.01
Myo-inositol	-0.71	< 0.01
Urea	-0.71	< 0.01
3-Methyl-2-oxovalerate	-0.75	< 0.01
